# Biomarkers of Chemotherapy-Induced Peripheral Neuropathy: Current Status and Future Directions

**DOI:** 10.3389/fpain.2022.864910

**Published:** 2022-03-14

**Authors:** Rozalyn L. Rodwin, Namrah Z. Siddiq, Barbara E. Ehrlich, Maryam B. Lustberg

**Affiliations:** ^1^Section of Pediatric Hematology/Oncology, Department of Pediatrics, Yale School of Medicine, New Haven, CT, United States; ^2^Section of Medical Oncology, Department of Medicine, Yale School of Medicine, New Haven, CT, United States; ^3^Department of Pharmacology, Yale School of Medicine, New Haven, CT, United States; ^4^Yale Cancer Center, New Haven, CT, United States

**Keywords:** chemotherapy-induced peripheral neuropathy (CIPN), paclitaxel, vincristine, bortezomib, biomarkers, oxaliplatin

## Abstract

Chemotherapy induced peripheral neuropathy (CIPN) is an often severe and debilitating complication of multiple chemotherapeutic agents that can affect patients of all ages, across cancer diagnoses. CIPN can persist post-therapy, and significantly impact the health and quality of life of cancer survivors. Identifying patients at risk for CIPN is challenging due to the lack of standardized objective measures to assess for CIPN. Furthermore, there are no approved preventative treatments for CIPN, and therapeutic options for CIPN remain limited once it develops. Biomarkers of CIPN have been studied but are not widely used in clinical practice. They can serve as an important clinical tool to identify individuals at risk for CIPN and to better understand the pathogenesis and avenues for treatment of CIPN. Here we review promising biomarkers of CIPN in humans and their clinical implications.

## Introduction

Chemotherapy induced peripheral neuropathy (CIPN) is a common and debilitating toxicity of cancer therapy. CIPN manifests with distal sensory and motor impairments, including pain, paresthesia, numbness, weakness, stiffness, and muscle atrophy (1), and can lead to impaired physical function and quality of life in cancer survivors (2–4). Patients at risk for CIPN range from children to adults, and span multiple cancer diagnoses (1, 5). Classes of chemotherapy implicated in CIPN include platinums, taxanes, vinca alkaloids, proteosome inhibitors, and angiogenesis inhibitors (5). As many a 68% of adult patients receiving neurotoxic chemotherapy develop CIPN, with one third of cases persisting post-therapy (6).

Despite the high prevalence and morbidity associated with CIPN, there are significant barriers to diagnosis and treatment. There is no standardized measure for CIPN, and current measures have limitations (7, 8). Objective measures including nerve conduction studies and the Total Neuropathy Score (TNS) can be invasive and time consuming, while patient-reported measures can be biased by subjective responses (8). Further, there are no approved treatments to prevent CIPN, and limited therapeutic options once it develops (9).

Biomarkers offer a novel approach to objectively identifying and risk-stratifying patients with CIPN and can provide insight into pathogenesis and treatment. Although studies of biomarkers of CIPN have increased over the past decade, they are still not part of routine clinical care. We present a review of promising biomarkers of CIPN in humans, and their implication for clinical care and future studies.

## Protein/Molecular Biomarkers of CIPN

**Figure 1:**
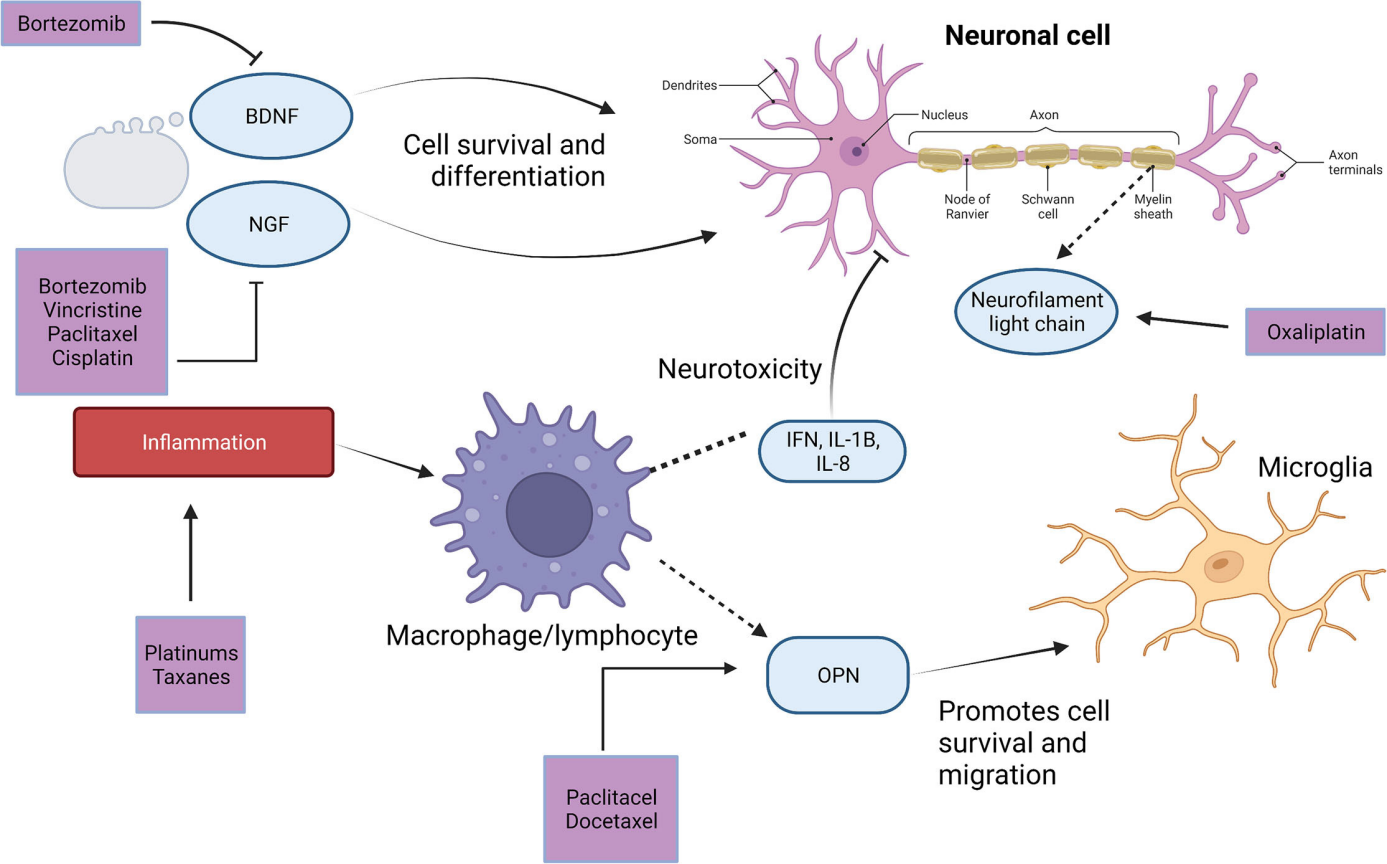
Serum markers of CIPN and their role in CIPN pathogenesis. Adapted from “Neuron Anatomy,” by BioRender.com (2022). Retrieved from https://app.Biorender.com/biorender-templates. CIPN, chemotherapy-induced peripheral neuropathy; BDNF, brain derived neurotrophic factor; NGF, nerve growth factor; OPN, osteopontin.

Increasing studies are identifying alterations in serum proteins and molecular markers in CIPN patients (Figure 1).

### Neurofilament Light Chain (NfL)

NfL is a neuronal cytoskeletal protein released with axonal damage (10). NfL was initially described as a marker of neurodegenerative diseases (11), and later of inherited neuropathies, and CIPN in animal models (10, 12).

Recently NfL has been studied in adults with CIPN. A prospective study of 43 patients receiving oxaliplatin evaluated serum NfL and CIPN severity by nerve conduction studies throughout therapy (13). Mean serum NfL levels increased over time, with significant differences in serum NfL between low grade (0–2) CIPN vs. high grade (≥3) CIPN at 6 months, and a cut-off of 195 pg/ml being 80% sensitive and 86.2% specific to identify high grade CIPN (13).

### Brain Derived Neurotrophic Factor (BDNF)

BDNF is a protein that promotes neuronal survival (14, 15). BDNF was associated with CIPN in 25 bortezomib-treated multiple myeloma patients evaluated for CIPN using the reduced Total Neuropathy Score (TNS-r) before and during therapy (16). Eight participants who developed CIPN had lower mean BDNF levels (2.16 ± 0.72 vs. 4.62 ± 0.61 ng/ml, *p* = 0.007), and were more likely to have a reduction from baseline BDNF (−1.67 ± 0.67 vs. 0.41 ± 0.71, *p* = 0.02) than those without CIPN (16). Similarly in 91 multiple myeloma patients treated with bortezomib or thalidomide lower BDNF levels during treatment were associated with CIPN by Common Terminology Criteria for Adverse Events (CTCAE), and a cut-point of 9.11 ng/ml was 76% sensitive and 71.4% specific to identify CIPN (17). Correlation between BDNF levels and CIPN by TNS-r was also reported in 22 non-Hodgkin lymphoma patients treated with vincristine (18).

Single nucleotide polymorphisms (SNPs) in *BDNF* may also confer increased risk for CIPN in individuals with Met/Met or Val/Met vs. Val/Val genotype (17–20). This association has been shown in bortezomib (16), and taxane-treated patients (20). A study of 35 breast cancer patients treated with taxanes, however, found the risk may be due to increased prevalence of baseline neuropathy, with no difference in prevalence of CIPN between genotypes when excluding patients with baseline neuropathy (21). There was also no association between the met-BDNF genotype and CIPN in 22 non-Hodgkin lymphoma patients treated with vincristine (18).

### Nerve Growth Factor (NGF)

NGF is a protein that also regulates neuronal survival (22). In 23 cancer patients receiving taxanes or platinums NGF levels decreased after four to six cycles of chemotherapy, and decline was associated with severity of CIPN by nerve conduction studies (23). Similarly in 129 plasma samples from 34 women treated for cervical cancer with paclitaxel and cisplatin, decrease in NGF from baseline was associated with CIPN severity by TNS (24). A prospective study of 45 patients with hematologic malignancies treated with bortezomib, thalidomide, or vincristine, also found there was a significant decrease in NGF in participants who developed CIPN symptoms, whereas there was no change in NGF in participants who did not develop CIPN symptoms (25). In contrast, in a study specifically evaluating neuropathic pain in 60 patients treated with platinum or taxane therapy, the 13 patients who developed painful neuropathy had higher NGF levels post-therapy than those without neuropathic pain (26).

### Osteopontin

Osteopontin is a glycoprotein involved in inflammatory pathways and has been implicated in cancer progression (27). It has been described as a marker of inflammation in other neurologic conditions included multiple sclerosis (28), and has also been implicated in neuronal repair (29). In a study of 50 breast cancer patients treated with taxanes evaluated by the TNS-r lower baseline levels of osteopontin were associated with developing moderate or severe CIPN, and baseline osteopontin levels were inversely associated with the magnitude of change in nerve conduction over time (30).

### Inflammatory Markers

The immune system has increasingly been implicated in CIPN in animal models (31, 32), but few studies examine the association of immune markers with CIPN in humans. In 67 breast cancer patients treated with taxanes there was a higher peripheral blood neutrophil-to-lymphocyte ratio in patients with CIPN than those without CIPN (33). In an analysis of cytokines in 55 breast cancer patients treated with taxane and platinum chemotherapy, high IFN-γ, IL-1β, and IL-8 and low IL-10 and IL-6 were associated with CIPN symptoms (34).

### MicroRNA, Proteomics, and Metabolomics

A recent approach to biomarker discovery in CIPN has included microRNA and exosome sequencing. In a preclinical model, miRNA-124 was associated with CIPN in mice treated with paclitaxel (35). MicroRNA may be a promising marker in humans as well, in cross-sectional analysis of microRNA expression in 84 breast cancer patients treated with paclitaxel, 15 microRNAs were identified with a significant fold change between CIPN and non-CIPN groups, and miRNA-451a was the most discriminatory (36). Mass spectrometry-based proteomic technology is another novel approach to biomarker discovery used to identify protein signatures associated with CIPN. In a study of 17 patients with breast cancer treated with taxanes, 12 protein signatures identified patients who developed CIPN (37).

## Genetic Biomarkers of CIPN

Genetic alterations are increasingly studied as predictors of disease toxicity. The following genetic alterations have been implicated in susceptibility to CIPN (Table 1).

**Table 1:** SNPs associated with CIPN sensitivity.

**Proposed Action**	**Gene**	**rsID**	**Associated Chemotherapy**	**CIPN Instrument**	**References**
Microtubule function	*CEP72*	*rs924607*	Vincristine	CTCAE, NCS	(38–41)
	*TUBB2A*	*rs9501929*	Paclitaxel	CTCAE	(42)
	*MAPT*	Additive SNPS	Paclitaxel, carboplatin	EORTCQLQ-OV28	(43)
	*GSK3B*	Additive SNPS	Paclitaxel, carboplatin	CTCAE	(43)
	*ACTG1*	*rs1135989*	Vincristine	CTCAE	(44)
	*CAPG*	*rs229668*	Vincristine	CTCAE	(44)
Drug metabolism/transport	*CYP3A5*	*rs776746*	Vincristine	CTCAE	(45)
	*CYP3A4*	*rs2740574*	Paclitaxel, docetaxel	CTCAE	(46)
	*CYP2C8*	*rs10509681*	Paclitaxel	CTCAE	(47)
		*rs1058930*	Paclitaxel	CTCAE	(42)
	*CYP1B1*	*rs1056836*	Paclitaxel	CTCAE	(42)
	*NR1I3*	*rs11584174*	Paclitaxel	CTCAE	(48)
	*UGT2B7*	*rs7662029*	Docetaxel	CTCAE	(48)
		*rs7438284*	Docetaxel	CTCAE	(48)
		*rs7439366*	Docetaxel	CTCAE	(48)
		*rs7668258*	Docetaxel	CTCAE	(48)
	*ABCB1*	*rs3213619*	Paclitaxel	CTCAE	(42)
		*rs4728709*	Vincristine	CTCAE	(44)
		*rs1128503*	Paclitaxel, docetaxel	CTCAE	(46)
		*rs1045642*	Paclitaxel	CTCAE	(49)
		*rs10244266*	Vincristine	WHO criteria	(50)
		*rs10274587*	Vincristine	WHO criteria	(50)
		*rs10268314*	Vincristine	WHO criteria	(50)
		*rs2032582*	Docetaxel, thalidomide	CTCAE	(51)
	*SLCO1B1*	*rs3829306*	Paclitaxel	CTCAE	(42)
	*ABCC1*	*rs2384937*	Bortezomib	Not specified	(52)
		*rs35604*	Bortezomib	Not specified	(52)
		*rs3887412*	Vincristine	NCI CTCAE	(53)
		*rs11864374*	Vincristine	WHO criteria	(50)
		*rs3743527*	Vincristine	WHO criteria	(50)
		*rs1967120*	Vincristine	WHO criteria	(50)
		*rs17501331*	Vincristine	WHO criteria	(50)
		*rs1293345*	Vincristine	WHO criteria	(50)
		*rs11642957*	Vincristine	WHO criteria	(50)
		*rs374867*	Vincristine	CTCAE	(40)
	*ABCC2*	*rs3740066*	Vincristine	WHO criteria	(50)
		*rs12826*	Vincristine	WHO criteria	(50)
	*ABCC6*	*rs8058696*	Bortezomib	Not specified	(52)
	*ABCG2*	*rs144018*	Oxaliplatin	CTCAE	(54)
	*PSMB1*	*rs1474642*	Bortezomib	CTCAE	(55)
	*DPYD*	*rs1413239*	Vincristine	CTCAE	(53)
Ion channels	*SCN9A*	*rs13017637*	Paclitaxel, docetaxel	CTCAE	(56)
		*rs6746030*	Oxaliplatin	TNS	(57, 58)
	*SCN4A*	*rs2302237*	Oxaliplatin	CTCAE	(59)
	*SCN10A*	*rs1262392*	Oxaliplatin	CTCAE	(59)
Inflammatory pathways	*FCAMR*	*rs1856746*	Paclitaxel	CTCAE	(60)
	*CTLA4*	*rs4553808*	Bortezomib	CTCAE	(55)
	*CTSS*	*rs12568767*	Bortezomib	CTCAE	(55)
	*IL17RD*	*rs1454981*	Bortezomib	Not specified	(52)
	*IL10RA*	*rs229113*	Bortezomib	Not specified	(52)
	*PSMB4*	*rs7172*	Bortezomib	Not specified	(52)
	*BTRC*	*rs4151060*	Bortezomib	Not specified	(52)
	*F2*	*rs31136516*	Bortezomib	Not specified	(52)
	*MBL2*	*rs216810*	Bortezomib	CTCAE	(53)
		*rs11003127*	Bortezomib	CTCAE	(53)
		*rs7071882*	Bortezomib	CTCAE	(53)
		*rs5030737*	Vincristine	CTCAE	(53)
	*PPARD*	*rs2267668*	Vincristine	CTCAE	(53)
		*rs7739752*	Bortezomib	CTCAE	(53)
		*rs6901410*	Bortezomib	CTCAE	(53)
		*rs6902123*	Bortezomib	CTCAE	(53)
		*rs6457816*	Bortezomib	CTCAE	(53)
Inherited neuropathies	*SBF2*	*rs149501654*	Paclitaxel	CTCAE	(61)
		*rs117957652*	Paclitaxel	CTCAE	(61)
		*rs141368249*	Paclitaxel	CTCAE	(61)
		*rs146987383*	Paclitaxel	CTCAE	(61)
		*rs7102464*	Paclitaxel	CTCAE	(61)
	*FZD3*	*rs7833751*	Paclitaxel	CTCAE	(61)
		*rs7001034*	Paclitaxel	CTCAE	(62)
	*FGD4*	*rs351855*	Paclitaxel, docetaxel	CTCAE	(46)
		*rs10771973*	Paclitaxel	CTCAE	(62)
	*ARHGEF10*	*rs9657362*	Paclitaxel	CIPN20	(63, 64)
		*rs2294039*	Paclitaxel	CIPN20	(63, 64)
		*rs1768288*	Paclitaxel	CIPN20	(63, 64)
Neuronal function	*TAC1*	*rs10486003*	Oxaliplatin	CTCAE	(65)
	*COCH*	*rs1045644*	Vincristine	CTCAE, TNS-PV	(66)
	*SOX10*	*rs139887*	Paclitaxel, carboplatin	FACT/GOG-Ntx	(67)
	*GPX7*	*rs3753753*	Paclitaxel, carboplatin	FACT/GOG-Ntx	(67)
	*NFATC1*	*rs9954562*	Bortezomib	Not specified	(52)
	*NFATC4*	*rs2228233*	Bortezomib	Not specified	(52)
	*EDN1*	*rs5370*	Bortezomib	Not specified	(52)
	*TCF4*	*rs1261134*	Bortezomib	CTCAE	(55)
	*DYNC1I1*	*rs916758*	Bortezomib	CTCAE	(55)
	*GJFE1*	*rs11974610*	Bortezomib	CTCAE	(55)
	*GNGT1*	*rs1858826*	Paclitaxel	CTCAE	(68)
	*EPHA4*	*rs17348202*	Paclitaxel, carboplatin	CTCAE	(69)
	*EPHA5*	*rs7349683*	Paclitaxel, carboplatin	CTCAE	(62, 69–71)
	*EPHA6*	*rs301927*	Paclitaxel, carboplatin	CTCAE	(69)
	*EPHA8*	*rs209709*	Paclitaxel	CTCAE	(71)
Cell cycle regulation/DNA repair	*CCNH*	*rs2230641*	Oxaliplatin	CTCAE, symptom reporting	(54, 72)
		*rs309816*	Oxaliplatin	Symptom reporting	(72)
	*ERCC3*	*rs2276583*	Bortezomib	CTCAE	(53)
	*ERCC4*	*rs1799800*	Bortezomib	CTCAE	(53)

*SNP, single nucleotide polymorphism; CTCAE, National Cancer Institute Common Terminology Criteria for Adverse Events; NCS, nerve conduction studies; EORTCQLQ-OV28, European Organization for Research and Treatment of Cancer Quality of Life Questionnaire-Ovarian Cancer Module; WHO criteria, World Health Organization criteria; CIPN20, European Organization for Research and Treatment of Cancer Quality of Life Questionnaire CIPN-20; TNS, Total Neuropathy Score; TNS-PV, Total Neuropathy Score-Pediatric Vincristine; FACT/GOG-Ntx, Functional Assessment of Cancer Therapy/Gynecologic Oncology Group-Neurotoxicity*.

### Genes Associated With Microtubule Function

Taxanes and vinca alkaloids interfere with microtubule function and may lead to CIPN pathogenesis (1, 73), therefore genes encoding microtubule function have been studied as predictors of CIPN sensitivity. An SNP in *CEP72 (rs924607)* is associated with CIPN in children and adults (38–41). In 48 adults with acute lymphoblastic leukemia (ALL) receiving vincristine, 75% with the TT genotype developed CIPN vs. 44% with CC or CT genotypes (39). In a combined sample of pediatric ALL patients treated with vincristine in two large therapeutic trials, the TT genotype was also associated with an increased risk for CIPN (38). This finding was replicated when measuring CIPN with nerve conduction studies (41), and in a separate cohort of pediatric ALL patients (40). However, other studies evaluating *CEP72* alterations did not find associations with CIPN in cohorts of Spanish and Arab patients treated with vincrisitne (74, 75). Additionally, there was no association of *CEP72* alterations with CIPN in patients treated with taxanes (43).

A polymorphism in *TUBB2A*, encoding tubulin, was associated with CIPN in 1,303 European patients treated with paclitaxel (42). However this finding has not been replicated in other studies of taxanes and vinca alkaloids (43, 76). Individual polymorphisms in *MAPT* (43, 76) and *GSK3B* (43) have not been associated with CIPN in patients treated with taxanes or vinca alkaloids, however additive polymorphisms in *MAPT* and *GSK3B* were associated with patient and clinician reported CIPN in 454 ovarian cancer patients treated with paclitaxel and carboplatin (43). SNPs in cytoskeletal protein genes, *ACTG1* and *CAPG*, have also been associated with CIPN in pediatric ALL patients treated with vincristine (44).

### Genes Associated With Ion Channels

Disturbance in neuronal function through ion channels may also contribute to CIPN, and alterations in these genes have been associated with CIPN sensitivity (1, 73, 77). In 186 Japanese breast and ovarian cancer patients treated with taxanes a SNP in *SCN9A*, encoding voltage-gated sodium channels, was associated with developing ≥grade 2 CIPN, and predicted CIPN persistence post-treatment (56). In 94 Spanish patients with gastrointestinal cancer treated with oxaliplatin another polymorphism in *SCN9A* was associated with a lower risk of acute CIPN by neurologic evaluation (57), and in 228 South Indian gastrointestinal cancer patients treated with oxaliplatin it was associated with increased incidence of chronic CIPN (58). In 200 patients with colorectal cancer treated with platinums, polymorphisms in *SCN4A* and *SCN10A* that encode voltage-gated sodium channels were associated with CIPN risk and severity (59). *SCN10A* has also been associated with chronic CIPN (58). Associations of mutations in voltage-gated potassium channels with CIPN have not been identified (78).

### Genes Associated With Inherited Neuropathies

Genes implicated in inherited neuropathies have also been examined in relation to CIPN. *SBF2*, associated with Charcot-Marie-Tooth disease, was associated with CIPN in 213 African American patients treated with paclitaxel (61). However, another prospective study of 58 paclitaxel-treated patients found *FZD3* was associated with CIPN, but not *SBF2* (61). In a study of 219 breast cancer patients treated with taxanes, *FGD4* was associated with CIPN (46). In a large prospective study of 855 patients of European Ancestry receiving paclitaxel, another polymorphism in *FGD4* was associated with patient-reported sensory CIPN, which was replicated in two additional cohorts (62). In the replication cohorts a different polymorphism in *FZD3* was also associated with sensory CIPN (62). *ARHGEF10* was associated with CIPN in a prospective study of 269 cancer patients treated in Alliance N08C1 that analyzed blood samples for 49 Charcot-Marie-Tooth genes (63). These findings were confirmed in 138 patients treated with paclitaxel in Alliance N08CA (64).

### Genes Associated With Inflammatory Pathways

There is a growing body of literature suggesting inflammation contributes to CIPN (73, 79, 80), and genetic alterations in inflammatory pathways have been studied in association with CIPN. In 3,431 breast cancer patients treated with paclitaxel a SNP in *FCAMR* that encodes the FC receptor, trended toward a significant association with CIPN (60). In 139 patients treated with bortezomib, variations in genes regulating immune function, *CTLA4* and *CTSS*, were associated with time to onset of CIPN, with a similar trend for *CTLA4* in a validation cohort (55). Bortezomib-neuropathy has also been associated with alterations in *IL17RD, IL10RA*, and genes in the NF-KB signaling pathway in 646 patients with multiple myeloma (52). Late-onset bortezomib-neuropathy was associated with polymorphisms in other genes in inflammatory pathways, *MBL2* and *PPARD* in 186 myeloma patients (53). In a meta-analysis of pediatric patients treated with vincristine for ALL from two large clinical trials *rs7963521*, associated with coding of the protein chemerin implicated in inflammatory pathways (66), was associated with CIPN.

### Genes Associated With Drug Metabolism and Transport

Polymorphisms in genes involved in chemotherapy metabolism may also increase CIPN sensitivity. In 107 children treated for ALL with vincristine *CYP3A5* polymorphisms were associated with CIPN (45). SNPs in *CYP2C8* and *CYP3A4* were associated with ≥grade 2 CIPN in two studies of breast cancer patients treated with taxanes (46, 47). In 79 breast cancer patients treated with taxanes SNPs in *NR1I3* and *UGT2B7* involved in drug metabolism were also associated with CIPN (48). In 1,303 patients treated with taxanes, additional SNPs in genes involved in taxane metabolism including, CYP2C8^*^4 and CYP1B1^*^3, were associated with CIPN, as were, *ABCB1* and *SLCO1B1*, involved in drug transport (42). In multiple myeloma patients treated with bortezomib, alterations in *PSMB1*, encoding drug binding proteins (55), and *ABCC1* and *ABCC6*, encoding drug transport, were also associated with CIPN (52). However, in a separate study of 369 multiple myeloma patients *ABCC1* polymorphisms were not associated with bortezomib-neuropathy, but were associated with vincristine-neuropathy, as was *DPYD* responsible for drug excertion (53). *ABCC1* polymorphisms have also been associated with CIPN in pediatric ALL patients treated with vincristine (40, 50). Alterations in, *ABCB1*, encoding drug transport, has also been widely associated with CIPN in patients treated with vincristine and taxanes (44, 46, 49–51), and alterations in *ABCC2* are associated with CIPN in children treated with vincristine (50). An alteration in *ABCG2*, involved in oxalate metabolism, was associated with oxaliplatin-induced neuropathy in 206 colon cancer patients (54).

### Other Genetic Alterations Associated With CIPN

Genes involved in nervous system development and function, and in cellular repair pathways, have also been associated with CIPN.

Alterations in genes encoding ephrin receptors *(EPHA4, EPHA5, EPHA6, EPHA8)*, a family of tyrosine kinase receptors involved in neural development, are associated with CIPN in patients treated with taxanes (62, 69–71, 81). An SNP in *TAC1*, encoding neuronal signaling hormones, was associated with CIPN in colon cancer patients treated with oxaliplatin (65). A polymorphisms in *COCH*, encoding cochlin involved in vestibular function and hearing loss, was associated with CIPN in a study of children with ALL treated with vincristine (82). Alterations in *SOX10*, involved in neuronal development, and *GPX7* were associated with CIPN in 107 survivors of gynecologic cancers treated with taxane or platinum (67). Polymorphisms in genes involved in nervous system function, *NFATC1, NFATC4*, and *EDN1* were associated with CIPN in 646 myeloma patients treated with bortezomib (52), as were *TCF4, DYNC1I1*, and *GJE1* in 139 myeloma patients treated with bortezmib (55). *GNGT1* encodes a protein in photoreceptors and has been associated with taxane-CIPN (68, 83).

Alterations in genes associated with DNA repair are also associated with CIPN. SNPs in *CCNH*, encoding cyclin H, involved in cell cycle progression and DNA repair (84), were associated with CIPN in 206 colon cancer patients (54), and in 228 gastrointestinal cancer patients treated with oxaliplatin (72). In myeloma patients treated with bortezomib, *ERCC4* and *ERCC3* involved in DNA repair were associated with CIPN (53). In a study of 680 testicular cancer survivors treated with cisplatin, lower expression of DNA repair gene *RPRD1B*, was associated with an increased risk of CIPN, which was replicated in two independent datasets (85).

## Pharmacokinetics and CIPN

Evaluation of drug pharmacokinetics may be another promising approach to identifying CIPN sensitivity.

### Taxane Pharmacokinetics

In 24 patients who received 12 weekly 3 or 1 h infusions of paclitaxel, longer duration of paclitaxel concentration >0.05 μM was associated with developing CIPN (86). In another prospective evaluation of 60 breast cancer patients receiving weekly paclitaxel infusions neither peak plasma concentration nor time above concentration of 0.05 μM were associated with CIPN, but were associated with increased toxicity-related treatment disruptions (87). An early study of lung cancer patients treated with paclitaxel also found no association between plasma concentration and neuromuscular or neurosensory outcomes (88).

### Vincristine Pharmacokinetics

An early study of pharmacokinetics in 54 children treated with vincristine did not find any association between vincristine clearance and neurotixicy (89). In a subsequent study assessing pharmacokinetics of vincristine in 74 pediatric patients, lower vincristine metabolite concentrations were associated with increased CIPN severity (45). Another study assessed vincristine pharmacokinetics in 35 patients receiving vincristine *via* push or 1 h infusions and found intercompartment clearance of vincristine was associated with an increased risk of CIPN, however other pharmacokinetic measures including maximum concentration were not associated with an increased CIPN risk (90).

## Discussion

We described promising biomarkers of CIPN in humans, including serum proteins, genetic polymorphisms, and drug metabolites. There are several limitations to the current studies and areas for future direction.

Serum protein biomarkers such as NfL, BDNF, NGF, osteopontin, and inflammatory markers have all been associated with CIPN, and may be easily translatable tools for detection and risk profiling in clinical practice (13, 18, 19, 24, 28, 91). However, these studies have been limited by small samples and variation in CIPN measurement between studies. Prospective validation studies of these biomarkers using objective CIPN measures would be helpful in confirming their clinical utility. Additionally, preclinical models should continue to be utilized to identify protein biomarkers that can be validated in humans.

Protein biomarkers can also inform therapeutic options that should continue to be explored. For example, in a study of 60 patients with bortezomib-neuropathy, patients randomized to receive NGF injections had better nerve conduction studies than those who did not receive NGF (92). In paclitaxel-treated rats, losartan had anti-inflammatory activity that resulted in lower inflammatory markers and decreased signs of CIPN (31). In addition to immune pathway targets, there are promising therapeutic targets that have been identified in critical CIPN pathways in animal models (73, 93). Neuronal Calcium Sensor-1 (NCS1), a protein involved in calcium signaling that binds taxanes and vinca alkaloids, decreases in CIPN in animal models (94–96). NCS1 may be a therapeutic target since lithium and ibudilast bind NCS1 and prevent CIPN in animal models (96), and retrospective studies show lithium may prevent CIPN in humans (97). Sterile alpha and TIR motif containing protein (SARM1) is another protein implicated in axonal degeneration in CIPN in preclinical models (73), and SARM1 inhibition may prevent CIPN (98, 99). Therefore, preclinical models can help better understand CIPN mechanisms, not only resulting in biomarker discovery that can be translated to the bedside, but also informing therapeutic strategies to prevent and mitigate CIPN that can be tested in humans.

Genetic polymorphisms are another avenue that offer promise in identifying individuals at risk for CIPN. *CEP72* has been identified as a risk factor for CIPN in multiple studies (38, 39, 43), and may help classify upfront risk for toxicity. Future studies should focus on whether treatment modification in at risk individuals alters toxicity and survival outcomes. Other genome wide studies identified numerous polymorphisms that may influence CIPN sensitivity, but few were replicated in multiple cohorts, therefore future studies should focus on replicating these findings. Another limitation of genome wide studies is that they only identify proteins with altered expression, however in other models altered function, rather than expression, of cellular components are proposed to initiate CIPN (93). Future studies should continue to elucidate CIPN pathogenesis through complementary mechanisms of genome wide studies and functional pathway analyses to identify therapeutic targets to mitigate this outcome.

Finally, pharmacokinetics is an evolving way to assess drug response and CIPN susceptibility in individuals receiving neurotoxic chemotherapy. Although current studies report mixed results regarding the ability to identify individuals at risk for CIPN (45, 87, 90), it warrants further exploration. Monitoring individual plasma drug concentration could offer a novel method to ensure adequate dosing for cancer treatment while minimizing risk for CIPN.

A limitation across studies is that there is no standardized method to define CIPN. We found most studies used CTCAE for CIPN grading, which lacks sensitivity and can vary by evaluator (100–102). Patient-reported outcome measures for CIPN have been validated in adults, and may be more sensitive for detection and measurement of change over time than the CTCAE (101–103). However, patient-reported outcomes still have limitations and do not always correlate with clinical assessments (104). It is important that future biomarker research incorporate robust, validated measures for CIPN that ideally combine patient-report and clinical evaluations (104).

Overall, there are many promising biomarkers of CIPN that can be valuable tools to aid in detection, risk stratification, and drug development. Future studies should prioritize large-scale validation of these biomarkers using standardized instruments to measure CIPN and expedite their implementation into clinical practice.

## Author Contributions

RR and ML contributed to concept, design, writing initial draft, editing final draft, and approval of final version. NS and BE contributed to writing initial draft, editing final draft, and approval of final version. All authors contributed to the article and approved the submitted version.

## Funding

RR was supported by the National Cancer Institute through the Yale Cancer Prevention and Control Training Program (T32 CA250803), as well as the Yale Pediatric Scholar Program, the William O. Seery Mentored Research Award for Cancer Research, Bank of America, N.A., Trustee, and Hyundai Hope on Wheels Young Investigator Award.

## Conflict of Interest

BE is a cofounder of Osmol Therapeutics, a company that is targeting NCS1 for therapeutic purposes. The remaining authors declare that the research was conducted in the absence of any commercial or financial relationships that could be construed as a potential conflict of interest. ML is a consultant for Osmol Therapeutics.

## Publisher's Note

All claims expressed in this article are solely those of the authors and do not necessarily represent those of their affiliated organizations, or those of the publisher, the editors and the reviewers. Any product that may be evaluated in this article, or claim that may be made by its manufacturer, is not guaranteed or endorsed by the publisher.
